# Safety around medicines for eye care

**Published:** 2021-07-20

**Authors:** Alaya Khatun, Victor H Hu

**Affiliations:** 1 Pharmacist (Ophthalmic and ePMA): York and Scarborough Teaching Hospital, York, UK.; 2 Assistant Clinical Professor: International Centre for Eye Health, London School of Hygiene & Tropical Medicine and Consultant Ophthalmologist, Mid Cheshire NHS Hospitals, UK.

**The medicine prescribing and dispensing process is complex, and errors are relatively common. This article looks at the various issues around prescribing and dispensing medicines for eye care and how patients can be kept safe.**

Many eye conditions are treated with some form of medicine, often requiring a prescription from an appropriately trained prescriber. The process is complex and errors can occur at any point along the pathway: from writing the prescription, to the patient instilling the correct eye drops, in the correct eye (as an example). Health care workers can and do make errors when writing prescriptions, and this has been well documented. One study in the UK showed that 7% of prescriptions contained errors.^1^ The researchers found that more experienced doctors, including consultants, were just as likely to make prescribing errors as their more junior colleagues. The same study showed that pharmacists intervened before most of the prescribing errors could affect patients.

**“The study found that more experienced doctors, including consultants, were just as likely to make prescribing errors as their more junior colleagues.”**

## Prescribing errors

Common issues or errors involving prescribing eye medicines include:

- **Wrong patient details added to a prescription.** This may result in confusion, delays, and patients being treated for a condition that they do not have (with a medication that may be unsafe for them) while others are left untreated for a condition that they do have.
- **Prescription written for the wrong eye.** For example, a patient has had surgery in their right eye but the topical steroid prescription is written for the left eye, leaving the patient without treatment to the eye that requires it, and potentially damaging the other eye.
- **Eye medications prescribed which are not available locally** (including those which are not on an agreed local formulary). This could include antimicrobial eye drops prescribed for a patient with a severe eye infection. Treatment for such conditions should start as soon as possible, but if the medication is not available locally the patient will not be able to start treatment in time.
- **Patient not advised about the expiry date of the product once it has been opened.** This can lead to treatment failure or worsening of the condition as a result of the patient using the preparation beyond the in-use expiry date.
- **Patient not advised about how to store the product appropriately.** This can lead to treatment failure or worsening of the condition as a result of the patient using the preparation after it has been stored incorrectly. Some eye drops should be refrigerated or kept cool; for example, chloramphenicol eye drops and ‘specials’ for more serious eye infections, such as amikacin and amphotericin B eye drops.
- **Preserved formulation supplied instead of preservative-free.** This can result in damage to the ocular surface. In some cases, this can worsen the condition that the patient is being treated for.
- **Wrong strength or dose.** For example, the strength of timolol eye drops is not specified on the prescription.
- **Poor communication with handover of prescribing responsibility.** This may lead to harm if, for example, patients are not issued continued prescriptions for a long-term treatment; e.g., if a community prescriber (such as a family practitioner) has received no communication from the hospital prescriber, and a patient on long-term glaucoma medication doesn’t receive their next bottle of eye drops after the one issued at the hospital runs out.

**Figure:**
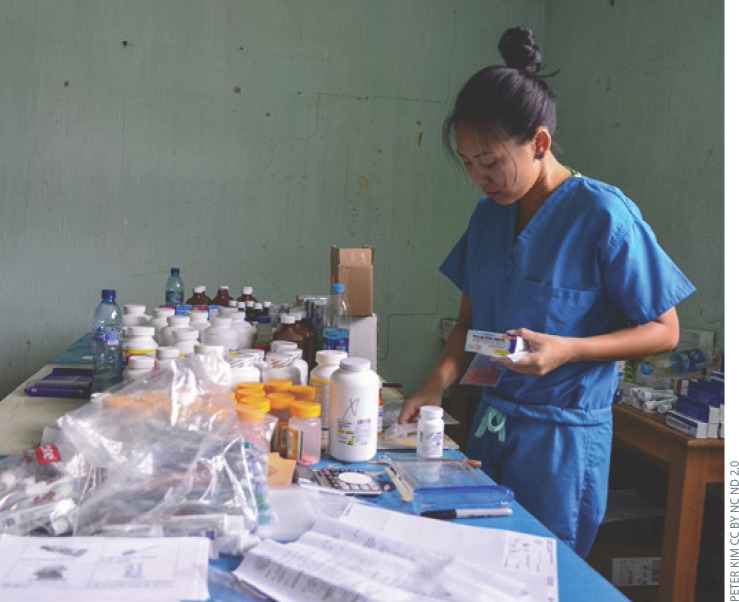
Pharmacists can protect patients by identifying and correcting prescription errors.

**Prescription writing checklist**

- Write legibly/clearly in ink so that the writing is indelible/permanent.
- Add the correct patient details, including the name, date of birth, and address.
- Include the prescriber details: name, signature, contact details, and medical registration (or equivalent).
- Include the date.
- Check any patient allergies.
- Use the generic name of the drug (unless there is a good reason to use a brand name, e.g., patient finds it easier to use the dispensing mechanism that comes with a particular brand).
- Include relevant medication information, including:
  - drug formulation, e.g., eye drop (bottle or single dose units), eye injection, tablets, etc.
  - dose, e.g., 1 drop.
  - frequency, e.g., four times a day.
- State which eye requires treatment, or if it is both.
- State the course duration or state that it must be used long-term.
- Follow locally approved formulary guidelines. This improves the patient’s ability to quickly access medicines, which is particularly important if delays in treatment could result in further significant damage to vision (e.g., in the case of hourly antimicrobial eye drops for microbial keratitis.
- Avoid abbreviations to help prevent delays and errors from misinterpretation, including the use of Latin and drug name abbreviations.

**Figure 1:**
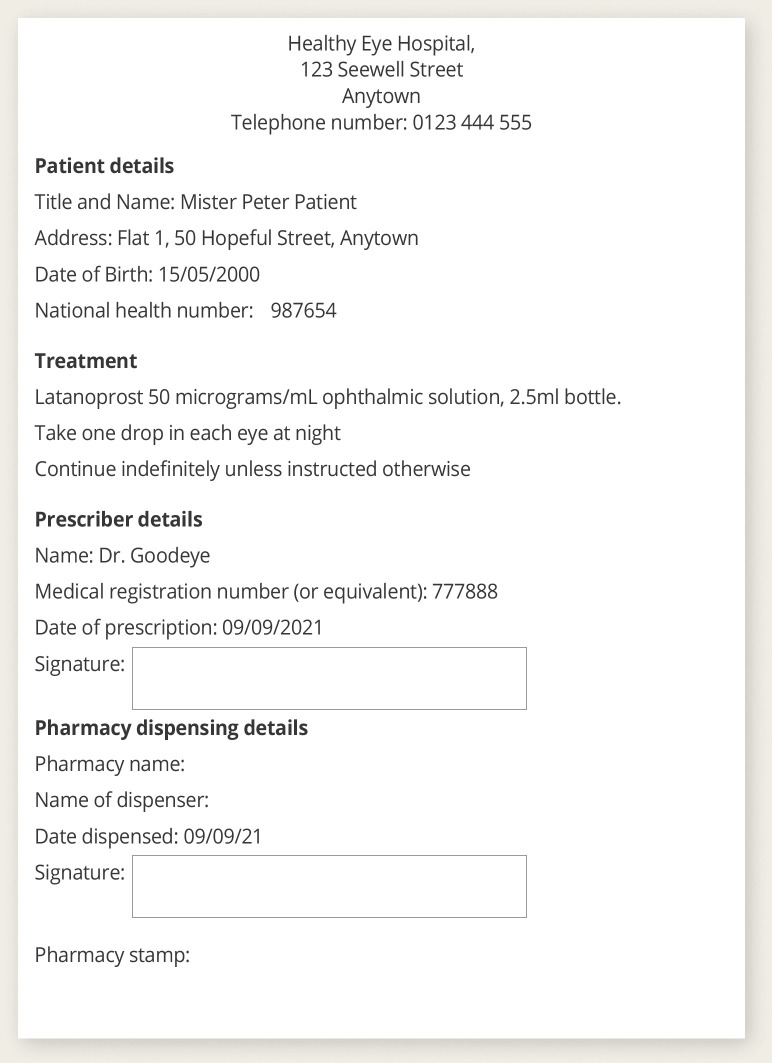
Example prescription containing all necessary information

## How to avoid prescribing errors

The majority of prescribing errors occur at the stage of writing the prescription. Prescribers can reduce the risk of these errors by checking and counter-checking each prescription before issuing it. A study from 2013 found that there were 4.7% (29/623) prescription errors during a one-month audit period. Following a process of counter-checking, this was reduced to just 0.77% (5/651).^2^

Therefore, prescribers should consider using a simple **prescription writing checklist** (see panel). These should meet at least the minimum legal requirements as well as other safety features, such as those recommended by the British National Fomulary:^3^

## Dispensing and administering medicines

Nurses and pharmacists have very important roles to play in preventing harm from prescription errors and ensuring that eye medications, including eye drops, are given correctly, so that patients gain the maximum therapeutic effect and avoid harm.

Pharmacists are the final point of contact in the community, before patients take their medicines home, and nurses are the final point of contact before patients are administered their medicines in hospitals.

They therefore need adequate knowledge and understanding of the relevant guidelines so they can identify potential errors and help patients to avoid them.

Consider the benefits of written information, in the form of patient information leaflets, to help with patient understanding. Where available, give these to patients when prescribing medications such as systemic steroids, as they can have severe and profound adverse effects which patients should be made aware of. Patients should also be given information about how to put in eye drops as this is something many patients find difficult. A previous article in this journal included a patient information leaflet on how to instil one’s own eye drops: **https://www.cehjournal.org/article/instilling-your-own-eye-drops/**

- Use a similar checklist to the one for prescribers (see panel) to help screen prescriptions for safety and appropriateness.
- As a safety precaution, two people should ideally check a medication before it is dispensed.
- If you are unsure what a medicine is being used for, what the correct dose is, or have any other concerns, then look it up in your local or national formulary and/or ask a colleague. If no local or national formulary is available, then a recognised standard can be used such as the British National Formulary (available online or in hard copy).^2^
- Develop the confidence to challenge prescribing decisions. Your ability to do this effectively could prevent patient harm. It may also help you to build mutual trust and productive working relationships with prescribing colleagues.
- Explain to the patient how they should put in their eye drops (or check that they know how to do this) and give them an instruction leaflet as a reminder (remember that some patients may not be able to read).
- Develop an effective mechanism for ensuring that the medicines you have in stock have adequate expiry dates. Arrange your stock according to expiry date and dispense those with the shortest expiry dates first. You can use paper- or computer-based spreadsheets to highlight when pre-packed kits such as crash boxes are about to expire.
- Eye drops, eye ointments, and eye gels have reduced expiry dates once opened, with many ‘specials’ having in-use expiry dates (expiry once opened) as short as 24 hours. Familiarise yourself with in-use expiry dates. This will also enable you to advise patients so that they can gain maximum benefit from the prescribed treatment.
- Become familiar with storage requirements of the medicines you work with to ensure that the integrity of stocked medicines is not compromised and that you are able to counsel the patient on the storage requirements.
- Hospital and non-hospital settings should be considered when administering topical eye medications.^4^ For instance, eye drops should be discarded after seven days and replaced if treatment continues in hospital settings. In non-hospital settings, eye drop bottles should be replaced every 28 days (or as directed by the information sheet).
- Nurses should ensure that the correct formulation of eye drops or eye ointment is administered at the correct time and strength, via the correct route, to the correct person, and into the correct eye.
- Nurses should advise patients against driving or operating machinery until their vision has cleared and/or their eyes have stopped stinging after administration of eye drops or ointment. This is particularly relevant when drops for pupil dilation are being instilled, as these can result in the vision being blurred for several hours.
- Nurses administering topical medication must keep careful record of topical drugs administration. Make accurate entries in the patients notes, as appropriate, in accordance with local and national guidance, e.g. those offered by the relevant professional body.
- Side effects and adverse reactions to medications should be documented in the patient’s notes and reported to the local/national reporting system, such as the Yellow Card scheme in the UK.^5^

**Figure:**
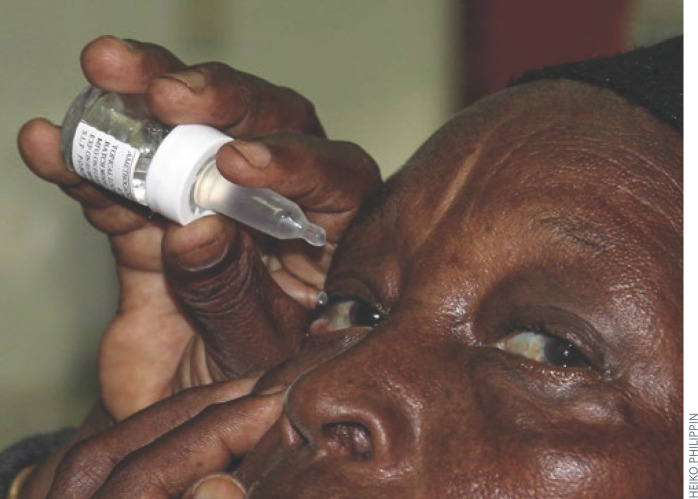
Show patients how to instil their own eyedrops. **TANZANIA**

**Paper-based prescribing vs electronic prescribing**

Many health care units benefit from electronic prescribing and medicines administration (ePMA) systems that mandate entry of the above information. Most ePMA systems also provide clinical decision support (information to help prescribers make the right decision), which is individualised for each patient, such as highlighting allergies.

ePMA systems have also been known to introduce problems alongside their numerous benefits. Prescribers have heightened expectations about what an electronic prescribing system can deliver, so are often surprised when they are found to have made an error that they expect an ePMA system would prevent. For example, not highlighting the need for a ‘once only’ loading dose for doxycycline prescriptions, leading to potentially slower onset of symptom resolution, or not mandating a review date or course length for steroid eye drop prescriptions, leading to inappropriate continuation of an acute prescription.

ePMA systems vary widely in terms of their level of sophistication. However, regardless of their digital maturity, you will still have to view your patient as a whole and exercise good clinical judgement when using the information they provide to care for your patients.
